# Feline congenital hypothyroidism: a case report

**DOI:** 10.29374/2527-2179.bjvm003423

**Published:** 2023-11-10

**Authors:** Gabriella Carvalho Abend, Stephanie Favato de Azevedo, Arthur Saturiano dos Santos, Gustavo Carvalho Cobucci, Heloisa Justen Moreira de Souza

**Affiliations:** 1 Veterinarian. Autonomus, Rio Grande do Sul, RS, Brazil; 2 Veterinarian, Programa de Pós-Graduação em Clínica Médica de Felinos, Centro Universitário de Tecnologia de Curitiba (UNIFATEC-PR), Polo Equalis Sul, Curitiba, PR, Brazil.; 3 Undergraduate in Veterinary Medicine, Instituto de Veterinária (IV), Universidade Federal Rural do Rio de Janeiro (UFRRJ). Seropédica, RJ, Brazil.; 4 Veterinarian, Autonomous, Centro de Diagnóstico por Imagem Avançada - Gamma Vet, Rio de Janeiro, RJ, Brazil.; 5 Veterinarian, DSc. Departamento de Medicina e Cirurgia Veterinária (DMCV), IV, UFRRJ. Seropédica, RJ, Brazil.

**Keywords:** scintigraphy, endocrinology, cat, levothyroxine, thyroid, cintilografia, endocrinologia, gato, levotiroxina, tireoide

## Abstract

We report a two-month-old male cat weighing 630 grams with congenital hypothyroidism. The main complaints were difficulty defecating for more than three days and prostration. Physical examination revealed a broad, flat face, a short neck, enlarged submandibular lymph nodes, chemosis, mild bilateral mucopurulent ocular discharge, seborrheic coat, with gingival thickening around the upper and lower deciduous incisor teeth with partial eruption. The abdomen was distended due to constipation and right unilateral cryptorchidism. Based on this, feline congenital hypothyroidism was suspected. Hormonal tests (free T4 by equilibrium dialysis of 0.06 ng/dl, total T4 of 0.1 ng/ml and TSH of 4.7 ng/ml) confirmed this. Treatment was started with levothyroxine sodium (5−32.2 µg/kg/day). After 120 days of treatment, there was clinical stabilization. Then the patient underwent orchiectomy of the left and of the right ectopic testicles, and at 380 days of treatment, the thyroid scintigraphy showed intense uptake of the radiopharmaceutical by both thyroid lobes and a significant increase in volume. Clinical evaluation showed weight gain (2.6 kilograms during treatment), improvement in the shape of the skull, and a notable increase in body size. At 17 months of age, hormone values were within the reference limits after administration of levothyroxine sodium (32.2µg/kg/day).

## Resumo

O objetivo deste trabalho foi relatar o caso de hipotireoidismo congênito em um felino, macho, pelo curto brasileiro (PCB), com dois meses de idade, pesando 630 gramas. Apresentava como queixa principal dificuldade para defecar por mais de três dias e prostração. O exame físico geral constatou face larga e plana, pescoço curto, linfonodos submandibulares aumentados, quemose e discreta secreção ocular mucopurulenta bilateral. A pelagem apresentava-se opaca e seborreica, espessamento gengival em torno dos dentes decíduos incisivos superiores e inferiores com parcial erupção. À palpação, o abdômen apresentava-se distendido pela presença de fezes em cólon e criptorquidismo unilateral direito. Diante das alterações supracitadas, suspeitou-se de hipotireoidismo congênito felino. O qual foi confirmado através de exames complementares hormonais específicos (T4 livre por diálise do equilíbrio 0,06 ng/dl, T4 total 0,1 ng/ml e TSH 4,7 ng/ml). Iniciou-se o tratamento com levotiroxina sódica na dose de 5 a 32,2µg/kg/dia. Com 120 dias de tratamento houve estabilização clínica e então o paciente foi submetido à orquiectomia dos testículos esquerdo ectópico e direito. E aos 380 dias de tratamento ao exame de cintilografia de tireoide onde foi evidenciado intensa captação do radiofármaco por ambos os lobos tireoidianos e importante aumento de volume. Na avaliação clínica, foi observado ganho de peso (2,6 quilogramas ao longo do tratamento), melhora na conformação do crânio e notável aumento de tamanho corporal. Aos 17 meses de idade, os valores hormonais se encontravam dentro dos limites de referência, após a administração da dose de lexotiroxina sódica na dose de 32,2µg/kg/dia.

## Introduction

Hypothyroidism is an endocrine alteration related to a decrease in the production of T3 (triiodothyronine) and T4 (thyroxine) by the thyroid, which is rare in felines. It usually occurs iatrogenically as a result of hyperthyroidism treatment, either surgically or with radioiodine therapy (Feldman et al., 2021). Hypothyroidism is classified as primary when it affects the thyroid, secondary when it occurs secondary to pituitary diseases, or tertiary when it occurs due to changes in the hypothalamus (Feldman et al., 2021).

Primary congenital hypothyroidism can be classified into thyroid dyshormonogenesis and dysgenesis. Thyroid dyshormonogenesis occurs when there is a defect in the thyroid hormone biosythesis (Jacobson & Rochette, 2018). Under these conditions, the negative feedback of the pituitary gland and hypothalamus is reduced, resulting in increased thyroid-stimulating hormone (TSH) secretion and hyperplasia or thyroid enlargement (goiter) (Stolf & Martins, 2016). In thyroid dysgenesis, there is thyroid hypoplasia or aplasia and the animal does not have goiter. Both cases may have a genetic (hereditary) origin (Feldman et al., 2021).

Clinical signs are identified in the first months of life, and include disproportionate dwarfism (large and broad head), short neck and limbs, constipation, megacolon, prolonged retention of deciduous teeth, and lethargy (Crowe, 2004). Diagnosis is made after analysis of the patient’s history, compatible clinical manifestations, laboratory changes, imaging tests, serum values of total T4, free T4 by balanced dialysis, and TSH (Greco, 2006). The treatment is through hormone replacement, with oral administration of levothyroxine at a recommended dosage range of 20 to 40 µg/kg/day every 24 hours, which can be adjusted according to the therapeutic response and total T4 values after the use of levothyroxine (Feldman et al., 2021).

Thyroid scintigraphy is a nuclear medicine test based on glandular radioisotope uptake that differentiates between thyroid dysgenesis and iodine defects in cats with hypothyroidism (Feeney & Anderson, 2007).

The objective of this report is to demonstrate the successful diagnosis and management of congenital primary hypothyroidism in a domestic cat, in addition to describing the use of thyroid scintigraphy as a means of diagnosing this pathology.

## Case report

**Figure 1:**
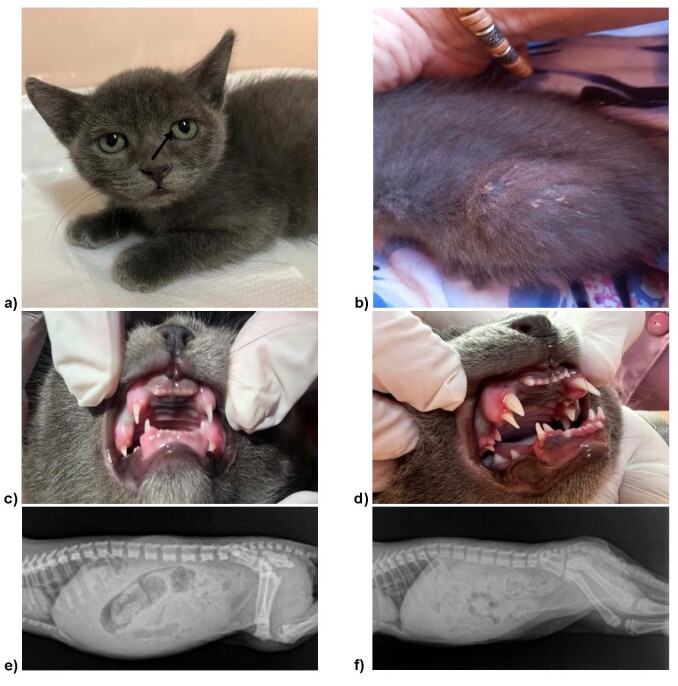
Patient with feline congenital hypothyroidism. **(A)** Wide flat face and short neck with small ears. **(B)** Note the dull coat and dry seborrhea. **(C)** Feline at 3 months of age with thickened gingival tissue covering the deciduous incisors (thin black arrow). **(D)** Feline at 10 months of age undergoing treatment with levothyroxine sodium with improvement of gingival tissue, presence of permanent teeth and retention of lower and upper right primary canine teeth. **(E)** A large amount of feces prior to treatment in the lateral abdominal X-ray. **(F)** At 4 months of age, there is late closure of the ossification centers.

A 2-month-old male Brazilian shorthair (BSH) cat, weighing 630 grams, was treated at a veterinary clinic specialized in pet cats, **Gatos&Gatos**. The main complaints were difficulty defecating for more than three days and prostration. The animal was rescued with another cat of the same litter (Figure 1A). The anamnesis revealed that the cat had received wet food for kittens and presented with paroxysmal sneezing and ocular and nasal discharge, compatible with feline viral respiratory complex.

Clinical examination revealed a wide and flat face, dense and seborrheic coat (Figure 1B), bilateral mucopurulent ocular exudation, chemosis, oral cavity with gingival thickening around the primary upper and lower incisors, with partial eruption (Figure 1C), ostensibly distended abdomen with a moderate accumulation of feces according to colonic topography, and a rectal temperature of 39.8° C.

Blood samples were collected for hematological examinations and biochemical profiling, and did not show significant alterations. Testing for feline viral immunodeficiency virus and feline viral leukemia was negative. The radiographic study in the right laterolateral, left laterolateral, and ventrodorsal projections revealed a large accumulation of feces in the colon, corroborating the initial suspicion of constipation (Figure 1E). The initial treatment was performed with the instillation of glycerin and warm water at a dose of 5 ml/kg with a number 10 urethral probe rectally and manual massage of the colon for the complete removal of feces. For this procedure, tranquilization was performed with intramuscular administration of methadone at 0.2 mg/kg. Support therapy followed with recombinant alpha-2-beta interferon at a dose of 50 IU/cat every 24 hours, amoxicillin with potassium clavulanate at 15 mg/kg orally every 12 hours for 7 days, lactulose syrup 667 mg/ml at a dose of 0.3 ml orally every 24 hours, gatifloxacin eye drops at 3 mg/ml (Zymar® eye drops) at one drop in each eye every 4 hours for 7 days. The inclusion of wet food for kittens and yogurt in the diet was recommended to facilitate the passage of feces.

The patient returned for reassessment one week after the first consultation and we found that there was no improvement in the constipation and the abdomen remained distended. The frequency of lactulose syrup administration was changed to every 12 hours and wet kitten food was continued.

After two weeks, the patient experienced a slight weight gain to 760 grams. According to the owner, the cat’s health had improved in relation to the previous condition of constipation, but the cat seemed to be indifferent to the environment. Upon clinical examination, palpation of the ventral cervical region revealed enlarged thyroid lobes (goiter), and right unilateral cryptorchidism. The radiographic examination revealed a delay in closing the ossification centers, marked epiphyseal lines in the long bones, irregularly formed vertebral bodies, and fecal retention (Figure 1F).

Feline congenital hypothyroidism was suspected. Therefore, new blood samples were collected for hematological examination, biochemical profiling, and measurement of serum cholesterol and triglycerides, with no noteworthy alterations. However, hormonal analysis demonstrated a free T4 value by equilibrium dialysis of 0.06 ng/dl (reference values by radioimmunoassay: 1.50 - 4.00 ng/dl) and a total T4 value of 0.1 ng/dl. ml (reference values by radioimmunoassay: 15-30 ng/ml), below the reference values, and a TSH value of 4.70 ng/ml (reference values by chemiluminescence: 0.03 - 0.38 ng/ml), above the reference values, as reported in Table 1.

**Table 1:** Hormonal test results and weight changes before and during levothyroxine treatment.

**Treatment time (days)**	**Total T4 (15 - 30ng/ml) Reference values**	**Free T4 by dialysis (1.50 - 4.00ng/dl) Reference values**	**TSH (0.03 -0.38ng/ml) Reference values**	**Levothyroxine dose (µg/kg/day)**	**Bory weight (kg)**
**0**	0.1	0.06	4.70	-	0.78
**30**	4.6	0.35	0.71	5	1.1
**90**	10	0.60	3.30	7	2.33
**120**	5.2	0.68	4.5	10	3.06
**150**	6.3	0.14	3.1	12	3.06
**180**	9.8	0.72	7	21	3.23
**360**	17.9	2.17	0.61	32.2	3.10

Sodium levothyroxine was prescribed at an initial oral dose of 5 µg/kg/day, one hour before meals for better drug absorption. This was prescribed for manipulated use owing to the ease and practicality of administration, given the difficulty of administering pills and the size of the patient. The patient returned after 30 days and showed a visible improvement in general condition. Seborrhea was absent, the coat was soft, the kitten had gained weight, and complete eruption of the deciduous teeth were observed. The animal wasnow interacting with the environment and with other cats. Defecation occurred daily and it no longer showed constipation or clinical signs of a feline viral respiratory complex. The hormone tests revealed improvement in relation to baseline levels, but still quite low, mainly free T4 and total T4. TSH levels were close to normal.

Monitoring was performed monthly for eight months with hormonal assessments and dose adjustments according to weight gain. The compounded levothyroxine doses were progressively increased to 7 µg/kg/day, 10 µg/kg/day, 12µg/kg/day, 21µg/kg/day and

**Figure 2:**
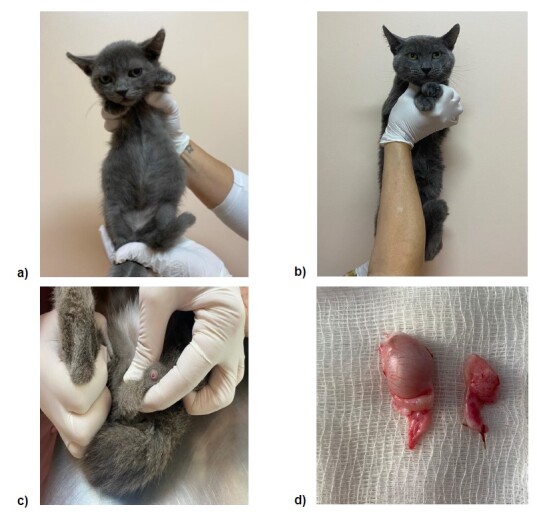
**(A and B)** Note the difference between the patient before treatment, with a lethargic appearance, rounded head and short stature, and after administration of levothyroxine sodium for one year. **(C)** Evidence of right unilateral cryptorchidism on palpation. **(D)** Excised testicles on the right through orchiectomy and on the left performed by abdominal laparotomy.

32.2µg/kg/day. At 10 months of age, the hormone levels were still not within the desired limits, possibly because of the initiation with low doses or inadequate administration. The clinical evaluation revealed a weight gain to 2.45 kg during treatment, improvement in the shape of the skull, the presence of permanent teeth, and a notable increase in body size (Figure 2A, 2B, 2C). However, the patient still had deciduous teeth (upper right and lower right and left canines) and right unilateral cryptorchidism (Figures 1D and 2D).

Abdominal ultrasound examination revealed that the left testicle was ectopic (located in the abdominal cavity), lateral to the bladder, next to the colon, measuring 1.20 x 0.57 cm, and with homogeneous parenchyma. Approximately three months after the examination, the patient was referred for an uneventful orchiectomy surgery. Due to the increase in dosage and the use of levothyroxine sodium (Synthroid®) at a dose of 32.2 µg/kg/day, the patient gained considerable weight, reaching 3.10 kg (Table 1).

One year after diagnosis, thyroid scintigraphy was performed with the subcutaneous application of 5 mCi technetium pertechnetate. Images were captured 60 minutes after application, with the animal manually restrained in the ventral and lateral positions. A Millenium GE gamma camera was used with a low-energy, high-resolution collimator for planar scintigraphy and a pinhole collimator for detailed images of the thyroid lobes. The following parameters were determined using scintigraphy: the percentage of technetium uptake, the thyroid/salivary gland ratio (T/S), the thyroid/background ratio (T/B) and the thyroid/heart ratio (T/H) (Broome, 2006; Peterson & Rishniw, 2021).

**Figure 3:**
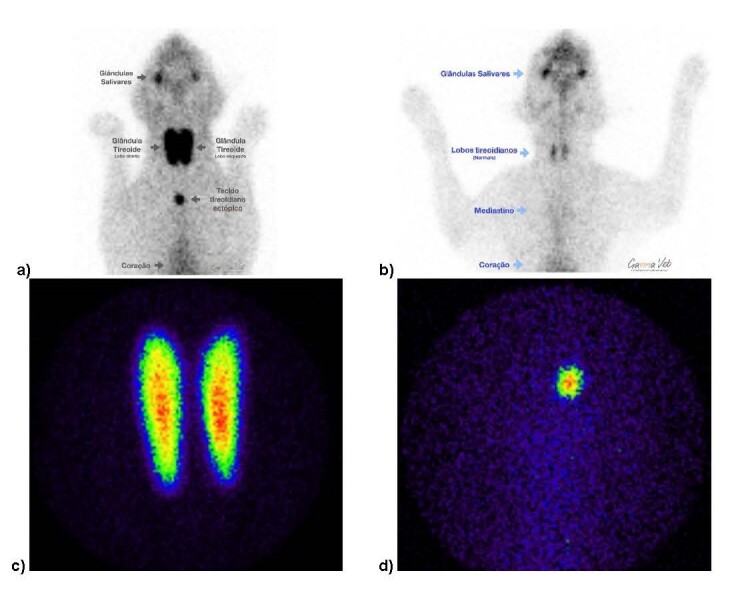
Thyroid scintigraphy in a 15-month-old cat in ventral and lateral positions. **(A)** Note that the thyroid lobes are increased in volume and with intense uptake of the radiopharmaceutical when compared to the salivary glands. Note the ectopic thyrodian tissue in the cranial mediastinum region. **(B)** Normal scintigraphy for comparison purposes only. **(C) (D)** Result of scintigraphic examination of both enlarged thyroid lobes and ectopic tissue using the pinhole method. The images are consistent with symmetric bilateral thyroid hyperplasia and ectopic thyroid tissue (hypothyroidism due to dyshormonogenesis).

The right and left thyroid lobes were enlarged, with a homogeneous appearance of radioisotope uptake and 8.83% technetium uptake (reference 0.05-0.8%) (Peterson et al., 2016). The T/S ratio was equal to 4.52 and 4.81 in the left and right lobe, respectively (reference <1.5). The T/B ratio was equal to 14.15 (reference <5.5) and the T/H ratio was equal to 5.69 (reference <1.5). Furthermore, ectopic thyroid tissue was observed in the cranial mediastinal region (Figure 3A, 3B, and 3C). The images were compatible with symmetric bilateral thyroid hyperplasia and ectopy of the thyroid tissue in the mediastinum, compatible.

## Discussion

Since the end of the 1980s, researchers from several academic centers have studied congenital hypothyroidism, which has been the subject of wide-ranging discussion regarding its prevalence and diagnosis.

Patients affected by congenital hypothyroidism are normal at birth, but developmental delay is noticeable in the first weeks of life, because thyroid hormones are essential for the normal development of the central nervous and skeletal systems. Primary hypothyroidism is related to the deficiency or inability to producethyroid hormones production (Quante et al., 2010). Pituitary (secondary) and hypothalamic (tertiary) causes of congenital hypothyroidism have not been reported.

Common clinical manifestations were observed in the reported patient, such as disproportionate size in relation to the littermates, lethargy, dry and seborrheic skin, and delayed tooth eruption. The growth retardation is due to epiphyseal dysgenesis and a delay in skeletal maturation, causing disproportionate dwarfism, characterized by a large and broad skull and jaw along with shortened ears (Peterson et al., 2018).

During the clinical evaluation of the patient, a distended abdomen was noted because of the large amount of feces, indicating severe constipation and megacolon. Gastrointestinal motility is often affected, however, the underlying mechanism remains unknown (Jacobson & Rochette, 2018; Quante et al., 2010).

Some researchers have reported imaging findings consistent with a delay in the closure of the epiphyseal plates of the long bones and vertebrae, as observed in the radiographic examinations of the patient in the present study (Feldman et al., 2021; Stolf & Martins, 2016). In British short-haired cats, closure of the ischiopubic plate was observed at two months ofage, and closure of the iliopubic and ilium-ischial plates at four months (Crowe, 2004). Radiography performed at four months revealed delayed closure of the ischiopubic plate.

Adequate levels of thyroid hormone are necessary for proper tooth development. Delayed eruption is a recurrent alteration in cats with hypothyroidism (Jacobson & Rochette, 2018). At three months of age our patient had thick gingival tissue covering the deciduous incisors, while after hormone replacement, at 10 months of age, the cat had all permanent teeth.

Dermatologic signs include retention of the youthful coat, thinning of the coat that progresses to alopecia with a lack of guard hairs, dry thickened skin, and changes due to a seborrheic and dull coat (Bojanic & Jones, 2011).

The palpable enlargement of the thyroid glands in our patient corresponded to another possible clinical finding of congenital hypothyroidism, suggesting thyroid dyshormonogenesis (Peterson, 2015).

The patient also exhibited cryptorchidism (right unilateral). In other reported cases, the alteration was bilateral, although its association with congenital hypothyroidism is unknown (Jacobson & Rochette, 2018; Quante et al., 2010).

In this report, the cat had a normal erythrogram, but anemia was expected, because the decrease in bone marrow stimulation by thyroid hormone precursors, including T4, often results in normochromic normocytic anemia (Stolf & Martins, 2016). The same can occur in the case of hypercholesterolemia and hypertriglyceridemia, which may be present according to serum cholesterol and triglyceride levels, since thyroid hormones are intrinsically linked to lipid metabolism (Bojanić et al., 2011). In this patient, both were values within normal limits. However, in hypothyroidism there is a decrease in lipid metabolism, leading to their accumulation in the plasma (Peterson et al., 2018).

A presumptive diagnosis can be made based on the clinical manifestations. However, the diagnosis should not be based solely on a low total T4 result, since many concomitant non-thyroidal diseases can also suppress the total serum T4 concentration, leading to a false-positive result (Peterson et al., 2001). We evaluated the serum thyroid profile, which included total T4, free T4, and TSH concentrations. The results showed low concentrations of thyroid hormones and high TSH levels, confirming primary hypothyroidism. Elevated TSH concentrations support the diagnosis of feline hypothyroidism, because falsely high TSH values are generally not observed in cats with non-thyroid disease (Galgano et al., 2014; Quante et al., 2010; Tanase et al., 1991).

Thyroid scintigraphy was performed to better define the diagnosis and differentiate thyroid aplasia or hypoplasia from dyshormonogenesis. In cats with thyroid aplasia or hypoplasia, no thyroid tissue is visible on scintigraphy, and radioisotope uptake by the thyroid is low or undetectable. In contrast, cats with dyshormonogenesis have increased technetium uptake in the thyroid gland, as documented in this report (Peterson, 2015; Peterson et al., 2018; Quante et al., 2010).

Stolf and Martins (2016) reported that hormone replacement with the synthetic thyroid hormone levothyroxine sodium is the treatment of choice. In humans, levothyroxine sodium acts as endogenous thyroxine and is converted into the active metabolite of T3 in the liver and kidneys (Geronimo et al., 2018).

In the present report, an initial oral dose of 5 µg/kg of levothyroxine sodium was administered every 24 hours (Crowe, 2004; Sjollema et al., 1991). This dose was effective for the initial control of the patient's clinical signs and there was a reduction in TSH, however it was not sufficient for free T4 and total T4 to approach normal values initially. After the patient's first evaluation, the dose was gradually adjusted to 7 µg/kg, 10 µg/kg/day, 12 µg/kg/day and 21 µg/kg/day, and 32.2 µg/kg/day, respectively, according to the weight gain and after new hormonal tests. In this sense, the indicated dose was adjusted to the range between 20 and 40 µg/kg, orally, every 24 hours, resulting in a good clinical result (Feldman et al., 2021; Golinelli et al., 2022). If resistance to levothyroxine supplementation occurs, with persistently high serum TSH concentrations, it may be useful to administer the dose on an empty stomach, as we did, as food can interfere with drug absorption (Feldman et al., 2021).

The objective of treatment was to increase the total T4 level to normalize serum TSH levels.

Notably as the kitten gains weight, the levothyroxine sodium dose should be adjusted (Feldman et al., 2021). The therapeutic response is fast, and after starting treatment, many clinical and laboratory parameters improve in one to three months, whereas dermatological and musculoskeletal signs have a slower resolution, of up to six months (Stolf & Martins, 2016). In our patient, the dermatological changes began to improve 15 days after starting the medication, along with considerable weight gain. Therapy should thus be monitored to enssure a favorable clinical response, along with the measurement of total T4, free T4, and TSH (Bojanić et al., 2011).

## Conclusions

Early diagnosis and the establishment of an effective treatment dose for hypothyroidism are important to prevent developmental delay and mental retardation in pet cats. In addition, scintigraphy and hormonal laboratory tests are important tools for diagnosis and follow-up treatment.
